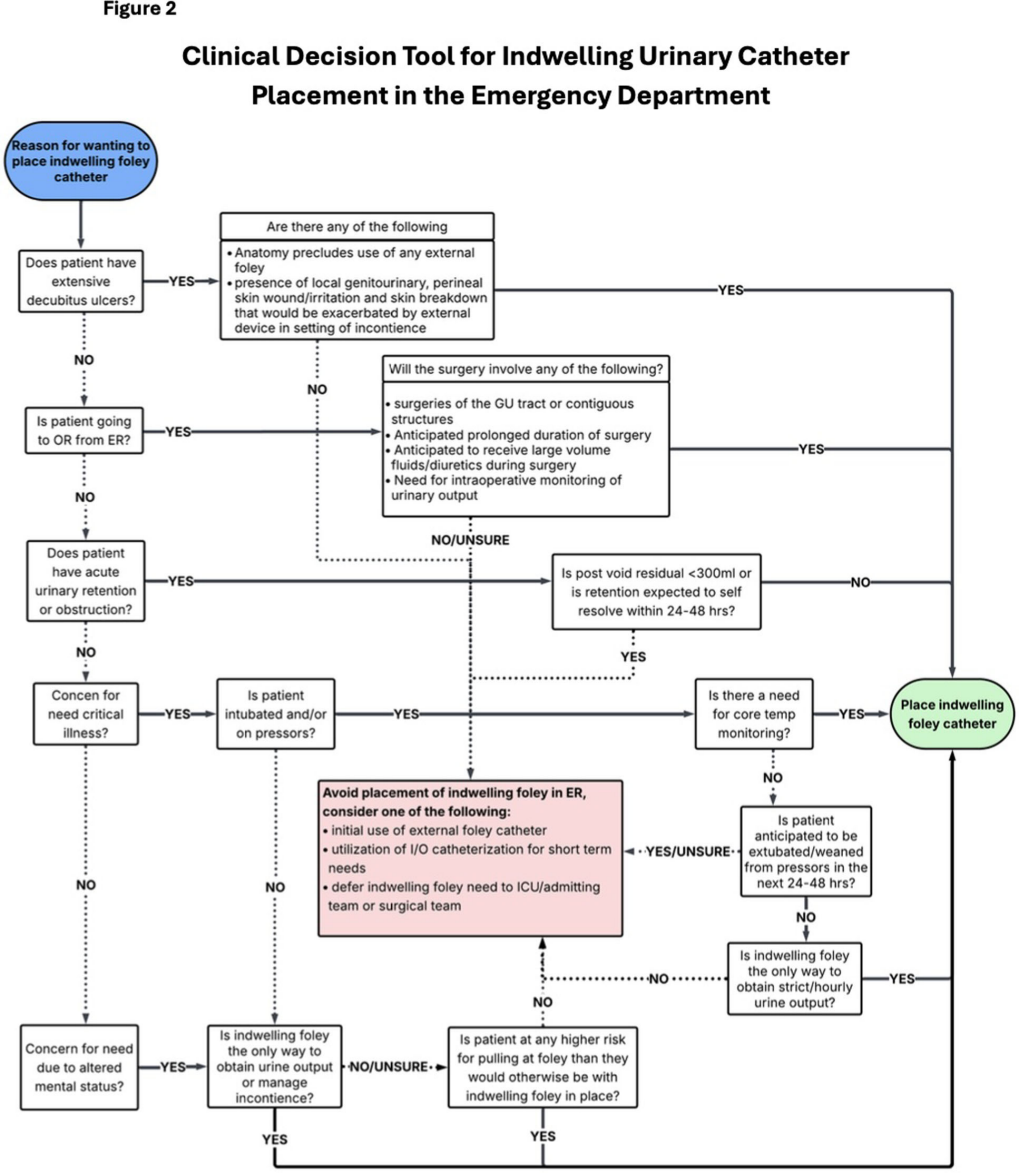# 146 Selective Pathogen Surveillance in Healthcare Water Systems: A Comparative Analysis of Ice Machines, Potable Water and Cooling Water

**DOI:** 10.1017/ash.2026.10551

**Published:** 2026-06-23

**Authors:** Jennifer Cihlar, Tom Talbot, Steven Katz, James Rickwa

**Affiliations:** 1 Vanderbilt University Medical Center; 2 Vanderbilt University School of Medicine; 3 Vanderbilt Wilson County Hospital

## Abstract

**Background:** Device stewardship is an important infection prevention opportunity for catheter-associated urinary tract infection reduction. This starts with the avoidance of initial catheter placement without definitive clinical need, especially with the option of alternative of non-invasive foley devices for both male and female anatomy. Urinary catheter placement in the Emergency Department (ED) is an area of opportunity where catheter placement could be avoided in favor of non-invasive management. This study assessed potential unnecessary catheter placement in a community hospital-based ED with a focus on ED-placed urinary catheters removed within 24 hours of admission. **Methods:** All patient encounters with an indwelling urinary catheter placed in 3 community hospital EDs from May 20, 2024 through September 29, 2025 were included. Encounters for patients who required chronic urinary catheterization (defined as the presence of a urinary catheter for ≥ 45 days) who had catheter replacement in the ED were excluded. The frequency of possible unnecessary catheter placement was defined as the number of catheters removed within 24 hours out of the total encounters. A subset of charts for encounters with a possible unnecessary catheter placed were reviewed to assess for commonalities to guide quality improvement. **Results:** During the study period, across all 3 facilities, 27.7% (385/1391) of urinary catheters placed in the ED were removed within 24 hours with a similar frequency at each individual facility (Hospital 1 = 24.6% (82/334), Hospital 2 = 24.9% (121/486), Hospital 3 = 31.9% (182/571)). The monthly frequency of possible unnecessary catheter placement ranged between 12.5% to 48.4% (Figure 1). Based on chart review, several common themes were noted, including placement in patients with altered mental status without hemodynamic instability and in patients with brief transient hemodynamic instability weaned from low dose inotropic medications and ventilation within 24 hours of admission. A clinical decision-making tool was created targeting recurrent scenarios that might warrant catheter avoidance (Figure 2). Discussion: ED placement of urinary catheters that were quickly removed by clinical teams upon admission was common across 3 community-based hospitals, highlighting possible unnecessary placement upon evaluation by the inpatient teams. A proposed clinical decision tool was created to help guide physicians in the reasoning behind the perceived need for catheter placement in the ED versus waiting to evaluate clinical need upon admission based upon patient course and failure of alternative methods for urine output management.

**Figure:**
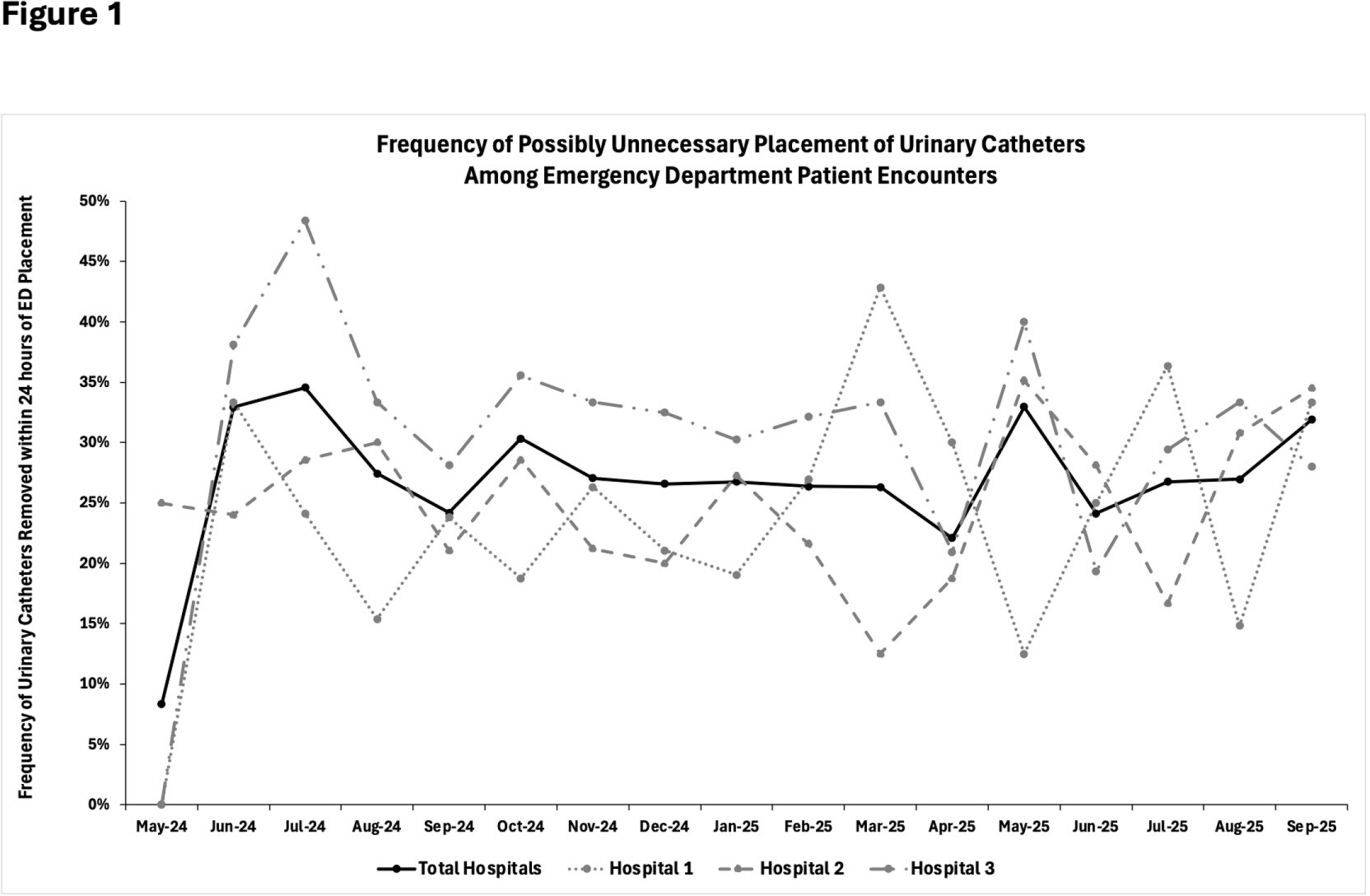

**Figure:**